# Improved linear growth after routine zinc supplementation in preterm very low birth weight infants

**DOI:** 10.1038/s41390-025-03935-z

**Published:** 2025-03-11

**Authors:** Tina A. Seidu, Luc P. Brion, Roy Heyne, L. Steven Brown, Theresa Jacob, Audrey Edwards, Cheryl S. Lair, Myra H. Wyckoff, David B. Nelson, Dimitrios Angelis

**Affiliations:** 1 Department of Pediatrics, University of Texas Southwestern Medical Center, Dallas, TX USA; 2 Children’s Health, Dallas, TX USA; 3 Division of Neonatal-Perinatal Medicine, Department of Pediatrics, University of Texas Southwestern Medical Center, Dallas, TX USA; 4 Parkland Health, Dallas, TX USA; 5 Department of Obstetrics and Gynecology, Division of Maternal-Fetal Medicine, University of Texas Southwestern Medical Center, Dallas, TX USA

## Abstract

**Background:**

This study was designed (1) to compare growth, morbidity and mortality in < 33-week gestational age (GA) (very preterm, VPT) or very low birth weight (BW < 1500 grams, VLBW) infants before (Epoch-1) and after implementing routine enteral zinc (Zn) supplementation (Epoch-2) to meet recommendations, and (2) to assess serum Zn levels and associated variables.

**Methods:**

Single-center prospective cohort of 826 infants. The primary outcome was the change (Δ) in Z-scores of accurate length (Δlength_z_), weight and head circumference from birth to discharge home.

**Results:**

In Epoch-2 vs Epoch-1 Δlength_z_ adjusted for confounding variables increased by 0.27 [95% confidence interval (CI) 0.13, 0.42, *P* < 0.001]. However, morbidity and mortality did not change. In Epoch-2 Zn decreased with GA and postnatal age: low ( < 0.74 mcg/mL) levels were observed in 51% infants. Retinopathy of prematurity (ROP) was independently associated with the lowest Zn [adjusted odds ratio 0.042 (CI 0.006, 0.306), area under the curve=0.928].

**Conclusion:**

Routine enteral Zn supplementation was independently associated with improved linear growth but did not prevent occurrence of low Zn. ROP was independently associated with the lowest Zn.

**Implications:**

Multicenter studies are needed to assess whether dosage of enteral Zn should be increased and whether Zn could help prevent ROP.

**Impact:**

- Implementation of routine enteral zinc (Zn) supplementation was associated with improved linear growth from birth to discharge and a more frequent physiologic growth curve in preterm very low birth weight infants.
- Serum Zn levels decreased with gestational age and with postnatal age.
- Low serum Zn levels were observed frequently despite routine Zn supplementation as currently recommended, which suggests a need to re-evaluate current enteral zinc supplementation guidelines for this population.
- Retinopathy of prematurity among infants < 33 weeks’ gestation was independently associated with low gestational age, low birthweight, stage of bronchopulmonary dysplasia and the lowest serum Zn level.

## Introduction

Preterm and very low birth weight (BW < 1500 grams, VLBW) infants are at risk of increased morbidity and mortality that can be, in part, mitigated by optimizing nutrition.^1^ Early and adequate nutrition is imperative to facilitate the healthy growth and development of these newborns, not only during the neonatal period, but also towards childhood and adulthood.^2–4^

Zinc (Zn) is the second most abundant micronutrient in the human body after iron and plays a vital role in cellular metabolism and functions. Infants with overt Zn deficiency develop growth failure with stunting, skin lesions, immune deficiency, and brain atrophy,^5–8^ but such severe Zn deficiency is less common in high-income countries. Since subclinical Zn deficiency is indolent, its detection requires a high index of suspicion and specialized testing. The frequency of subclinical Zn deficiency in premature newborns is underestimated and could affect up to 30–50% of preterm newborns,^9^ with significant effects, mainly related to the brain, the gastrointestinal (GI) tract and immune functions.^10,11^

Although serum Zn level has limitations in its interpretation and represents only a small fraction of the total body Zn pool, it remains the most common way of assessing Zn status in newborns. While there is significant variation in normative serum Zn levels with postnatal age, a range of 0.74–1.46 mcg/mL (11.3–22.3 mcmol/L) has been recommended for preterm infants.^12^

Recent expert opinion recommendations and nutritional societies favor an increase in daily Zn supplementation in preterm infants (enteral Zn 2–3 mg/kg/day, parenteral Zn 400–500 mcg/kg/day).^12–18^ Systematic review of randomized controlled trials (RCTs) has shown that Zn supplementation [enterally and in parenteral nutrition (PN)] in preterm infants may decrease all-cause mortality and improve weight gain and linear growth but not other outcomes.^19^

Several conditions contribute to Zn deficiency in premature newborns. Maternal Zn deficiency is prevalent in several low-income countries^20^ and in association with specific conditions (smoking, malabsorption, vegan diet, etc.) can contribute to low maternal stores and possibly low fetal Zn transfer.^21,22^ Since most fetal accretion of Zn occurs in the second and third trimesters, preterm infants are at risk for Zn deficiency. After birth, poor absorption of Zn in the immature GI tract, insufficient enteral or PN intake and urine loss all contribute to Zn deficiency. Donor human milk (DHM) used to supplement mother’s own milk (MOM) could be an additional factor for poor Zn provision.^10^ In a previous study from our group, among very preterm ( < 33wks GA, VPT)/VLBW infants who consumed MOM and/or DHM, 14% were diagnosed with Zn deficiency associated with growth failure pattern.^23^

Gaps in knowledge are as follows. 1. The optimal dosing, timing and duration of enteral Zn supplementation for VPT/VLBW infants is not known. 2. Higher than recommended dose of Zn supplementation appears to improve necrotizing enterocolitis (NEC), feeding intolerance and growth in limited studies, when started early in life, but it is unclear if the currently recommended dose has any significant effect on these morbidities. 3. Zn is a key micronutrient for normal retinal function, but its effect on retinopathy of prematurity (ROP) is unclear.^24^

The current study was designed to address some of the above gaps in knowledge: (1) to compare growth in VPT/VLBW infants before (Epoch-1) and after (Epoch-2) implementing routine enteral Zn supplementation to meet recommendations, (2) to describe changes of serum Zn levels with GA, postnatal age (PNA) and postmenstrual age (PMA), to identify variables associated with serum levels and (3) to compare morbidity and mortality in the two epochs, with emphasis on the relationship between Zn and ROP. The primary hypothesis was that growth parameters – defined by changes in Z-scores of accurate weight, length and fronto-occipital head circumference (FOC) and growth patterns– would improve after implementing routine Zn supplementation. Secondary hypotheses were that after Zn supplementation, in Epoch-2, rates of neonatal morbidities, such as NEC and ROP would improve, when compared to Epoch-1.

## Methods

### Design

**Fig. 1:**
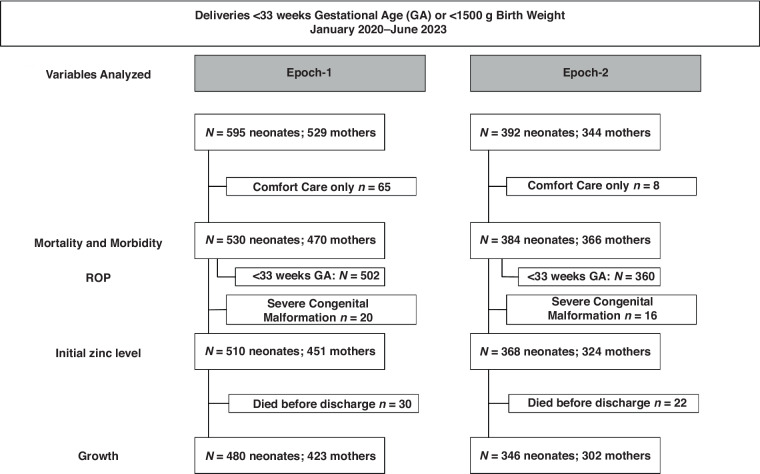
Flow diagram.

This was a prospective cohort study with pre-test, post-test analysis of infants born between January 1st, 2020, and June 30th, 2023, in a single institution. Infants included in this study were born VPT or VLBW before (Epoch-1, January 2020-December 2021) and after (Epoch-2, January 2022–June 2023) implementing routine enteral Zn supplementation to meet current recommendations and routine measurements of serial Zn levels in the NICU. Growth was analyzed in a subgroup of this cohort, excluding infants who received comfort care only, were diagnosed with a congenital or chromosomal anomaly that could impact growth, or died prior to discharge (Fig. 1). The data used for infants in Epoch-1 was previously published.^23^ Inclusion of new infants was stopped before implementation of new feeding guidelines in July 2023.

### Setting

Parkland Health and Hospital System (PHHS) is the county hospital in Dallas, TX. It serves a predominantly Hispanic and non-Hispanic Black population, > 85% covered by Medicaid. All inborn VPT/VLBW neonates are admitted to a 96-bed Level III neonatal intensive care unit (NICU).

Enteral feedings were provided using specific feeding guidelines and a feeding adjustment protocol based on growth patterns and serial accurate measurements.^25^ Iron was provided as iron sulfate at a dose of 3–4 mg/kg/day elemental iron, starting at 2 weeks (2wks) of age or when reaching full feeds, with no changes in dosing throughout the period of the study. A DHM program was established at PHHS in January 2020. The DHM supplied by the Donor Milk Bank of North Texas was 20 kcal/oz and contained 1 g/dL of protein as measured by infrared spectroscopy. DHM was provided with signed consent to infants of mothers with insufficient MOM supply. These infants were eligible for DHM until 36wks PMA, and then transitioned to preterm formula if MOM supply was insufficient.

### Data collection

Maternal and neonatal demographic information was obtained from the electronic health records (EHR) and from validated NICU, Maternal-Fetal Medicine, and neonatal resuscitation databases. The Parkland NICU database has been approved by the UTSW IRB as a quality improvement initiative.^26^

Maternal data included race/ethnicity, ZIP code, medical insurance, pre-pregnancy or first trimester body mass index (BMI), pregnancy complications, such as gestational hypertension and diabetes mellitus, prenatal care, magnesium sulfate administration, and delivery method. Census tract-based material community deprivation index was extracted using ZIP code tabulation area to ZIP conversion from publicly available data.^27,28^ Neonatal data included GA, anthropometric measurements at birth, Apgar scores, delivery room interventions, temperature at admission, accurate length measurements, hyperglycemia ( > 150 mg/dL or >8.3 mmol/L), and morbidity and mortality in the NICU. Serial measurements for weight, length, and FOC were obtained from the EHR. An algorithm described previously was used to retrieve additional nutritional information from the EHR, such as enteral feedings, protein and energy intake, total duration of PN and number of days to reach full feeds (120–160 ml/kg/day).^23^

Growth was primarily assessed by the change (Δ) in Z-scores of accurate length, weight, and FOC from birth to discharge home as defined previously.^29,30^ Registered Dietitians in PHHS’s NICU were trained in obtaining accurate and precise length measurements using a caliper or length board.^29^ Serial weights were analyzed in the EHR for the first four weeks of life and compared to patterns by Rochow et al. to determine if the infant had either physiologic, excessive weight loss, or excessive weight gain based on GA.^30^ Growth patterns from four weeks of life until discharge were analyzed in the EHR and classified into physiologic growth ( < 1 Z-score decrease in weight and/or length), insufficient growth (poor weight and length), insufficient/excessive weight gain, insufficient linear growth, transient poor growth (resolving before discharge), or other.^29^

### Zinc measurements and zinc supplementation

In Epoch-1, infants received 450 mcg/kg/day of Zn in PN. Serum Zn level was obtained in infants with excessive decline in length Z-scores or weight and length Z-scores from birth despite using the adjustable feeding protocol.^25,29^ Additionally, a serum Zn level was obtained in those with risk factors for Zn deficiency, such as short bowel syndrome. Infants with a documented Zn deficiency (serum Zn level < 0.74 mcg/ml) received 3–3.5 mg/kg/day enteral supplementation. Extremely low BW infants ( < 1 kg) on PN with documented Zn deficiency received an additional 50 mcg/kg/day in PN (total of 500 mcg/kg/day in PN). Average growth in Epoch-1 was similar to the period before implementing the DHM program.^23^ However, energy and protein intake increased by 5% and the frequency of Zn and vitamin D supplementation increased by 30%.

In Epoch-2, VPT and VLBW infants were provided with 500 mcg/kg/day of Zn in PN, or 650 mcg/kg/day if Zn-deficient. Once at 140 ml/kg/day of enteral feeds, infants received routine enteral Zn supplementation (as Zn Chloride) to achieve a total of 2.5 mg/kg/day until 36wks PMA or discharge; total intake was slightly increased by 0.3–0.5 mg/kg/day (to a total dose of 2.7-3 mg/kg/day) on July 1, 2022 based on new recommendations.^16^ Serial Zn levels were obtained at birth, when enteral feeding volume was 140 ml/kg/day (full feeds), and one month after the second level. Infants with Zn deficiency received a total daily dose of 3.5 mg/kg/day Zn, which was adjusted based on serum level within a month of Zn supplementation.

In both epochs, enteral Zn was administered separately (at another feeding time) from other trace elements to optimize their absorption. Copper (Cu) supplementation was provided to infants receiving Zn supplement at doses yielding a 10:1 (weight:weight) Zn:Cu ratio. Blood samples (0.6 ml) for Zn measurements were collected in navy blue metal-free tube and centrifuged. The Zn level in non-hemolyzed serum samples was measured at the Mayo Clinic using Dynamic Reaction Cell-Inductively Coupled Plasma-Mass Spectrometry.

### Outcome variables

The primary outcome was growth assessed by change in Z-scores from birth to discharge home in accurate measurements of length (Δlength_z_).

Secondary growth outcomes included changes in Z-scores of weight (Δweight_z_) and FOC (ΔFOC_z_), initial weight curve during the first 4wks postnatal, subsequent growth pattern and average weight gain calculated using Patel’s geometric method.^31^ Additional outcomes included serum Zn levels, Zn deficiency, vitamin D deficiency/insufficiency, feeding tolerance, hyperglycemia, mortality to discharge and morbidity outcomes: retinopathy of prematurity (ROP),^32^ BPD,^33^ late onset sepsis, gastrointestinal perforation, spontaneous intestinal perforation (SIP), necrotizing enterocolitis (NEC) stage II or greater^34^ and severe intraventricular/periventricular hemorrhage (IVH) (grade 3 or 4).^35^

Serum 25-hydroxy (OH) vitamin D level was obtained in infants with poor growth as defined above. The level of 25-OH vitamin D was measured at the Mayo Clinic laboratory via Liquid Chromatography Tandem Mass Spectrometry, with a volume of 0.5 ml/sample. With this technique errors secondary to measurement of false or similar metabolites, or epimers are minimized.^23,25,36,37^ Vitamin D insufficiency (serum level < 30 ng/mL) or deficiency ( < 20 ng/mL) was treated with enteral cholecalciferol to achieve a total of 2000 units/day in 4 doses.^23^ A repeat serum level was measured within a month.

### Statistical analysis

Statistical analysis was done using SPSS version 29 (IBM Inc, Armonk, NY) or SAS version 9.4 (SAS Institute, Inc, Cary, NC). Numbers are presented as mean ± standard deviation (SD), median and interquartile range (IQR) or number (%). Normality was assessed using the Kolmogorov-Smirnov test and the Shapiro-Wilk test. For variables that did not follow normal distribution, non-parametric tests were applied. Continuous variables were analyzed using Student t-test, Mann-Whitney test; analysis of variance adjusted for GA, PMA at discharge, and sex; quadratic regression analysis with confidence and prediction intervals; analysis of variance by rank; nonparametric analysis of covariance; generalized linear regression; or mixed model general linear regression analysis by rank. Independent association of Δlength_z_ with epoch was assessed by generalized linear regression adjusted for race/ethnicity, antenatal steroids, sex, GA, protein and energy intake and morbidity that could affect growth. Dichotomous variables were analyzed using chi-square analysis or Fisher’s exact test followed by pair-wise comparisons with Bonferroni correction; binary or multinomial logistic regression; or stepwise forward logistic regression analysis.

Post-hoc analysis included the development of predictive models for ROP or severe ROP [stage ≥ 4, anti-vascular endothelial growth factor (VEGF) therapy or laser therapy]. Model performance was determined by (a) discrimination assessed by the area under the curve (AUC) of the receiver operating characteristic (ROC) curve and compared by using the SAS logistic procedure with ROC contrast; (b) goodness of fit assessed by the calibration curve and the Hosmer-Lemeshow test.^38,39^ The calibration plot was obtained using the SAS logistic program with a locally weighted least squares regression smoother (i.e., the loess algorithm) and a value of 0.75 for the span parameter.^40^ Models were internally validated using bootstrap samples with SPSS to determine whether bias-corrected accelerated 95% confidence interval (CI) of the b coefficients were different from zero.

Statistical significance, set at *P* < 0.05, was not adjusted for multiple testing. All tests were performed on complete data and two-tailed.

Internal validation of the NICU database was done by comparing data extracted from EHR for this study on 5 variables: GA, BW, NEC, SIP, and severe IVH in extremely low GA neonates (< 29wks, ELGANs). Statistical analysis for validation included intraclass correlation coefficient for continuous variables and Cohen kappa for dichotomous variables.

Sample size analysis was based on the hypothesis that implementation of Zn supplementation would be associated with Δlength_z_ of 0.25, based on a previous study that our group published previously.^23^ Based on initial results of Δlength_z_ during the first 6 months of Epoch-2, we estimated that 684 infants (384 in Epoch-1 and 300 in Epoch-2) would be sufficient to detect a difference in Δlength_z_ from −1 to −0.75 with a standard deviation (SD) of 1, as previously described,^23^ using a Student t-test with a power of 0.9 and an alpha error of < 0.05. We planned to include 725 infants to adjust for exclusions at birth and death prior to discharge.

### Approval

The Institutional Review Board of University of Texas Southwestern Medical Center and Parkland Health approved the study and waived the need for individual consent.

## Results

### Demographics

From 987 infants in total cohort, mortality and morbidity were analyzed in 914, after excluding 65 infants in Epoch-1 and 8 infants in Epoch-2 who received comfort care only (Fig. 1). Growth and nutritional intake were analyzed in the growth cohort, 480 infants in Epoch-1 and 346 infants in Epoch-2, after excluding 50 infants in Epoch-1 and 38 infants in Epoch-2 for severe congenital anomaly diagnosis, or death before discharge (Fig. 1). The maternal and neonatal characteristics of the growth cohort (Table 1) were similar in both epochs, except for prenatal/first trimester BMI, sex and admission temperature. Late cord clamping was applied to 20 neonates in Epoch-2 and none in Epoch-1.

**Table 1:** Comparison of baseline maternal and neonatal characteristics in the growth cohort in both epochs.

Characteristics	Epoch-1	Epoch-2	*P-*value
Maternal	*N* = 423	*N* = 302	
Prenatal/first trimester maternal BMI (kg/m^2^)	32.50 ± 7.18 (366)	31.00 ± 8.23 (199)	0.03
Multiple gestation	54 (12.8%)	44 (14.6%)	0.48
Material community deprivation index > 0.3	316/422 (74.9%)	235/299 (78.6%)	0.25
Medicaid	375 (88.7%)	272 (90.1%)	0.55
Prenatal care	399 (94.3%)	279 (92.4%)	0.30
Pregnancy-induced hypertension	190 (44.9%)	128 (42.4%)	0.50
Diabetes mellitus	63 (14.9%)	56 (18.5%)	0.19
Antenatal steroids	380 (89.8%)	265 (87.7%)	0.38
Magnesium sulfate	240 (56.7%)	166 (55.0%)	0.45
Race/ethnicity			*0.27
Hispanic	282 (66.7%)	189 (71.6%)	
Non-Hispanic Black	117 (27.7%)	62 (23.5%)	
Non-Hispanic White	20 (4.7%)	8 (3.0%)	
Other	4 (0.9%)	5 (1.9%)	
Cesarean delivery	268 (63.4%)	206 (68.2%)	0.18
**Neonatal**	***N*** = **480**	***N*** = **346**	
Female	224 (46.7%)	187 (54.0%)	0.04
Gestational age (weeks)	30.7 (28.3, 32.1)	30.8 (28.3, 32.1)	0.95
Gestational age <30 weeks	196 (40.8%)	139 (40.2%)	0.85
Birthweight (grams)	1403 (1090, 1690)	1430 (1030, 1720)	0.85
Weight Z-score	0.12 ± 1.18	0.08 ± 1.18	0.65
Arterial cord blood pH	7.25 ± 0.09 (314)	7.24 ± 0.09 (217)	0.62
Arterial cord blood base deficit (mmol/L)	−6.7 ± 4.1 (313)	−6.8 ± 3.9 (217)	0.71
Delayed cord clamping	-	20 (5.8%)	-
Apgar score at 5 min	8 (7,9)	8 (7,9)	0.46
Maximum F_i_O_2_ in delivery room	0.40 (0.30, 1.00)	0.40 (0.30, 1.00)	0.14
Endotracheal intubation in delivery room	56 (11.7%)	37 (10.7%)	0.66
Chest compressions in delivery room	4 (0.8%)	0 (0.0%)	0.14
Admission temperature (degrees centigrade)	36.3 ± 0.8	36.1 ± 0.7	< 0.001
Admission temperature			< 0.001
36.5–37.5 °C	158 (32.9%)^a^	78 (22.7%)^b^	
< 36.5 °C	300 (62.5%)^a^	259 (75.5%)^b^	
> 37.5 °C	22 (4.6%)^a^	6 (1.7%)^b^	
Small for gestational age	58 (12.1%)	41 (11.8%)	0.92
Birth FOC (cm)	27.5 (25.5, 29.0)	27.5 (25.0, 29.5)	0.95
FOC Z-score	−0.07 ± 1.03	−0.06 ± 1.09	0.92
Accurate length in 1^st^ week (cm)	38.7 (35.7, 40.7) (334)	38.8 (35.0, 41.2) (259)	0.51
Accurate length Z-score	−0.29 ± 1.94	−0.39 ± 0.88	0.36
BMI using first accurate length (kg/m^2^)	8.37 (7.56, 9.19) (334)	8.50 (7.54, 9.30)	0.44
BMI Z-score	−0.43 ± 1.42	−0.45 ± 2.24	0.86

Values are mean ± SD (n if less than N in top row), median (IQR) or number (%).

Student t-test, Mann-Whitney test, χ^2^ analysis or *Fisher’s exact test.

^a,b^Different alphabetic superscripts across columns indicate significant pair-wise differences.

*BMI* body mass index, *F*_*i*_*O*_*2*_ fractional inspiration concentration of oxygen, *FOC* fronto-occipital circumference.

When baseline characteristics of neonates in the growth cohort are compared to neonates with a congenital anomaly or who experienced death, the growth cohort infants were born at a lower GA (29.8 ± 2.9wks vs 30.8 ± 2.8wks, *P* = 0.04), had a lower BW Z-score (0.06 ± 1.21 vs −0.38 ± 1.3, *P* = 0.04), lower birth FOC Z-score (−0.10 ± 1.12 vs -0.59 ± 1.31, *P* = 0.01) and higher median 5-minute Apgar score (8 vs 7, *P* = 0.03) (Supplementary Table 1). Accurate length was available in the first week in 688/826 infants (83.2%).

### Nutrient intake, Delta Z-scores for growth parameters and growth patterns

The percentage of infants receiving MOM did not change between the 2 epochs, whereas the percentage receiving DHM increased from Epoch-1 to Epoch-2 (Table 2a). There was no significant difference in total energy and protein intake between the two epochs (Table 2a). Enteral Zn supplementation started earlier in Epoch-2, at a median PMA of 32 wks (ELGANs) and 33wks (GA ≥29wks), versus 34 and 38wks, respectively, in Epoch-1 (*P* < 0.001) (Table 2a).

**Table 2:** Nutrition and growth characteristics in the growth cohort among epochs. a. Comparison of enteral feeding, energy and protein intake, and serum zinc levels in both epochs in the growth cohort. b. Comparison of growth and size at discharge in the growth cohort in both epochs.

a				
Variable	Number 826	Epoch-1 *N* = 480	Epoch-2 *N* = 346	*P*-value
**Any donor human milk**	826	392 (81.7%)	322 (93.1%)	< 0.001
**Any maternal own milk**	826	428 (89.2%)	298 (86.1%)	0.19
**Only formula**	826	7 (1.5%)	1 (0.3%)	*0.15
**Any maternal own milk at discharge**	826	227 (47.3%)	163 (47.1%)	0.96
**Days to full feeds** (120–160 ml/kg/d)	808	3 (3, 4)	3 (2, 4)	< 0.01
**Duration of PN**, median days	818	41 (29, 61)	42 (28, 61)	0.34
**PMA when enteral Zn supplementation started (weeks)**	596			
**22**–**28 weeks GA**	375	34.1 (33.4, 36.0) (63)	32.0 (30.1, 33.1) (312)	< 0.001
≥ **29 weeks GA**	221	37.7 (34.4, 39.4) (9)	32.7 (31.9, 33.6) (212)	< 0.001
**Average total intake first 4 weeks of life**				
Energy(kcal/kg/d)	818	105.4 (96.8, 111.4)	106.1 (97.6, 111.1)	0.73
Protein (g/kg/d)	817	3.1 (2.7, 3.3)	3.1 (2.7, 3.3)	0.23
**Average total intake after 4 weeks of life**				
Energy (kcal/kg/d)	638	127.9 (121.5, 136.1)	127.4 (120.3, 137.5)	0.82
Protein (g/kg/d)	637	4.0 (3.7, 4.4)	4.0 (3.6, 4.4)	0.26
**Average enteral intake, median (IQR)**				
Energy (kcal/kg/d)	816	112.1 (103.3, 118.8)	111.7 (105.1, 118.9)	0.82
Protein (g/kg/d)	816	3.5 (3.1, 3.7)	3.4 (3.1, 3.7)	0.42
**Average intravenous intake, median (IQR)**				
Energy (kcal/kg/d)	816	16.7 (13.6, 20.0)	16.9 (14.4, 20.2)	0.24
Protein (g/kg/d)	516	1.4 (1.1, 1.8)	1.5 (1.2, 2.0)	< 0.01
Average weight gain* (g/kg/d)	826	10.7 ± 2.8	10.9 ± 2.6	0.27
**Any serum zinc level**	826	73 (15.2%)	328 (94.8%)	< 0.001
**Lowest serum zinc level (mcg/mL)**	401	0.56 (0.51, 0.68) (73)	0.73 (0.64, 0.84) (328)	< 0.001
**Initial serum zinc level (1st** **48** **h) (mcg/mL)**	218	N/A	0.98 (0.85, 1.19) (218)	NA
**Latest serum zinc level (mcg/mL)**	310	N/A	0.76 (0.67, 0.88) (310)	NA
**b**				
**Growth Characteristics**	826	***N*** = **480**	***N*** = **346**	
Initial weight curve during 1^st^ 4 weeks	811	*N* = 467	*N* = 344	*0.18
Physiologic		343 (73%)	272 (79%)	
Excessive weight loss		121 (26%)	70 (20%)	
Excessive weight gain		3 (1%)	2 (1%)	
Subsequent growth curve pattern	815	*N* = 473	*N* = 342	*< 0.001
Physiologic		187 (40%)	204 (60%)	
Growth failure		53 (11%)	35 (10%)	
Linear growth failure		50 (11%)	32 (9%)	
Weight loss		68 (14%) ^a^	9 (3%) ^b^	
Excessive weight gain		6 (1%)	0 (0%)	
Other or not applicable		109 (23%)	62 (18%)	
PMA at discharge (weeks)		36 (34, 39)	36 (35, 38)	0.46
Weight (g)		2533 (2250, 2905)	2560 (2270, 2951)	0.37
Weight Z-score		−0.84 ± 0.90	−0.83 ± 0.85	0.87
PMA at length measurement (weeks)		36 (34, 39)	36 (35, 38)	0.46
Accurate length (cm)		46.1 (44.8, 48.0)	46.2 (44.6, 48.0)	0.79
Length Z-score		−1.07 ± 1.20	−1.11 ± 1.09	0.65
Body mass index (kg/m^2^)		11.6 (10.8, 12.8)	11.7 (11.0, 12.8)	0.61
Body mass index Z-score		−0.31 ± 2.02	−0.20 ± 0.85	0.34
Fronto-occipital circumference (cm)		33 (32, 34)	33 (32, 34)	0.88
Fronto-occipital circumference Z-score		−0.37 ± 0.92	−0.44 ± 0.98	0.31

Values are number of infants/total number of infants (%), median (IQR) (n if number < N in upper row) or mean ± SD.

Student t-test, Mann-Whitney test, or *Fisher’s exact test

Different alphabetic superscripts across columns indicate significant pair-wise differences.

*DBM* donor breastmilk, *MOM* mother’s own milk, *PN* parenteral nutrition, *PMA* postmenstrual age, *IQR* interquartile range, *GA* gestational age

*From birth to discharge, calculated using Patel geometric method

Δlength_z,_ available in 659 infants (96% of expected and 79.8% of the growth cohort), changed significantly from Epoch-1 to Epoch-2 (−0.94 ± 1.21 vs −0.70 ± 0.83, respectively, *P* = 0.002). However, there were no notable differences in the change in Z-scores for weight or FOC and no changes in size at discharge or Z-scores of size at discharge. When adjusted for GA, PMA at discharge and sex, average adjusted Δlengthz improved by > 20% from Epoch-1 to Epoch-2 (*P* < 0.001) **(**Table 3a). Multivariable analysis showed that Δlengthz adjusted for confounding variables increased by 0.27 [95% confidence interval (CI) 0.13, 0.42, *P* < 0.001] in Epoch-2 (Table 3b).

**Table 3:** Change in Z-scores from birth to discharge in infants < 33 weeks or < 1500 g in the growth cohort during both epochs. a. Changes in weight, length and FOC. b. Change in length Z-scores: multivariate analysis.

a							
Variable	Unadjusted^a^			Adjusted for GA, postmenstrual age at discharge and Sex^b^			
	Epoch-1	Epoch-2	*P-*value	Sex	Epoch-1	Epoch-2	*Epoch P-*value
Delta WeightZ	−0.96 ± 0.74 *N* = 479	−0.91 ± 0.76 *N* = 346	0.34	Females Males	−0.94 ± 0.05 −1.00 ± 0.05	−0.88 ± 0.05 −0.94 ± 0.05	0.27
Delta LengthZ^c^	−0.94 ± 1.22 *N* = 351	−0.70 ± 0.83 *N* = 308	0.003	Females Males	−0.91 ± 0.06 −1.00 ± 0.06	−0.65 ± 0.06 −0.74 ± 0.07	< 0.001
Delta FOCZ	−0.29 ± 0.93 *N* = 470	−0.39 ± 0.95 *N* = 340	0.13	Females Males	−0.23 ± 0.06 −0.35 ± 0.06	−0.32 ± 0.06 −0.45 ± 0.07	0.19

b		
Variable	Beta Coefficient (95% Confidence interval)	*P-*value
Epoch-2 vs Epoch-1	0.27 (0.13, 0.42)	< 0.001
Gestational age (by week)	0.23 (0.20, 0.26)	< 0.001
Antenatal steroids	−0.07 (−0.30, +0.17)	0.58
Male	−0.11 (−0.26, +0.03)	0.13
Race ethnicity		
Hispanic	(reference)	-
Non-Hispanic Black	0.05 (−0.11, +0.22)	0.54
Non-Hispanic White	0.24 (−0.15, 0.63)	0.23
Other	0.17 (−0.47, +0.82)	0.60
Severe bronchopulmonary dysplasia^d^	0.11 (−0.24, +0.45)	0.55
Culture-positive late onset sepsis	0.09 (−0.27, +0.44)	0.64
Gastrointestinal perforation	−0.17 (−1.49, +1.14)	0.80
Average energy intake (cal/kg/day)^e^	−0.02 (−0.03, −0.01)	0.001
Average protein intake (cal/kg/day)^e^	0.53 (0.24, 0.83)	< 0.001

^a^ mean ± standard deviation Student t-test; ^b^ mean ± standard error four-way ANOVA

^c^Accurate measurements obtained by dietitian with length board or caliper within the first week of life and at discharge.

*GA* gestational age, *Delta []*_*Z*_ change in Z-score from birth to discharge for [], *FOC* fronto-occipital circumference

Generalized linear equation, *n* = 621

^d^NIH consensus definition

^e^during the entire hospitalization

There was no statistical difference in growth pattern in the first four weeks between Epoch-1 and Epoch-2 (Table 2b). Subsequent growth curve pattern was significantly different between Epoch-1 and Epoch-2 (*P* < 0.001), respectively: physiologic (40% vs 60%, *P* < 0.05), growth failure (11% vs 10%), linear growth failure (11% vs 9%), weight loss (14% vs 3%), and other (23% vs 18%),

### Initial Serum Zn level (First level within the 48 hours postnatal)

**Fig. 2:**
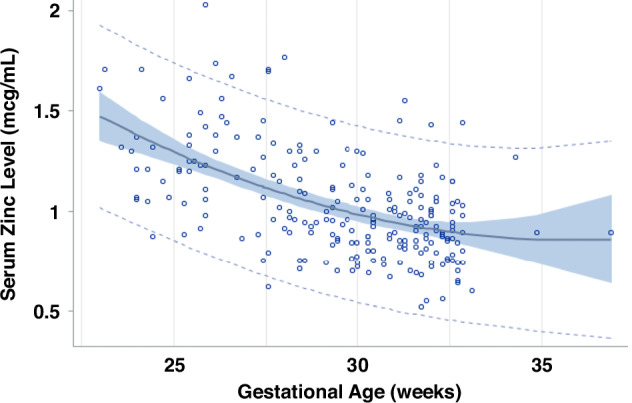
Early Zinc levels (first 48 hours of life) by gestational age. 95% confidence and prediction intervals and residuals of first zinc level during the first 48 h of life versus gestational age among infants < 33 weeks gestational age or < 1500 g birth weight in Epoch-2, excluding those without congenital anomalies or comfort care only. Quadratic regression analysis. Adjusted R^2^ = 0.2921, *n* = 228, *P* < 0.0001. Equation: Zn = 5.63727 −0.26622 X GA + 0.00370 x GA^2^. Zn Serum zinc level, GA gestational age (weeks).

The first Zn level was obtained with 48 h postnatal in 226 infants (14% products of multiple pregnancy) among the subgroup of 368 infants born in Epoch-2, excluding infants who received comfort care only or were diagnosed with a congenital or chromosomal anomaly (Fig. 1). The initial level decreased with an increase in GA (Fig. 2); this decrease fitted best a quadratic regression; the formula (Table 4a) was used to estimate the expected level for GA. In multivariable analysis, the ratio of initial serum Zn level to expected level for GA was significantly lower in association with race/ethnicity (other vs Non-Hispanic Black, non-Hispanic White and Hispanic), high material community deprivation index, high maternal BMI, and exposure to antenatal steroids, and higher in association with prenatal care. It was not associated with medical insurance, sex or SGA (Table 4b).

**Table 4:** Initial serum zinc level: Serum zinc level within the first 48 hours among infants born in Epoch-2 excluding infants on comfort care only and those with severe congenital anomalies. a. Quadratic normative regression vs gestational age. b. Multivariate analysis of the ratio of initial serum zinc level to GA-expected value from quadratic regression versus prenatal and early neonatal variables.

a			
Variable	Beta coefficient (standard error)	95% Confidence interval	*P*-value
Gestational age (by week)	−0.2620 (0.1065)	−0.4708, −0.0532	0.014
Gestational age squared	+0.0036 (0.0018)	0.00002, 0.0072	0.049
Intercept	+5.580 (1.5309)	2.679, 8.580	< 0.001

b			
Variable	Beta coefficient (standard error)	95% Confidence interval	*P*-value
Race/ethnicity			< 0.001
Hispanic	Ref	Ref	Ref
Non-Hispanic Black	0.030 (0.036)	−0.040, +0.100	0.40
Non-Hispanic White	−0.098 (0.109)	−0.311, +0.116	0.37
Other	−0.248 (0.038)	−0.321, −0.174	< 0.001
Maternal BMI (kg/m^2^)^a^			0.03
< 18.5	Ref	Ref	-
18.5– < 25	−0.445 (0.169)	−0.776, −0.113	0.009
25– < 30	−0.385 (0.168)	−0.714, −0.057	0.02
$\ge 30$	−0.357 (0.166)	−0.683, −0.032	0.03
Material community deprivation index > 0.30	−0.107 (0.042)	−0.189, −0.026	0.01
Prenatal care	+0.208 (0.039)	0.132, 0.285	< 0.001
Pregnancy-induced hypertension	0.100 (0.036)	0.030, 0.170	0.005
Antenatal steroids	−0.175 (0.045)	−0.263, −0.087	< 0.001
Intercept	+1.376 (0.176)	1.032, 1.721	< 0.001

Generalized linear equation, *n* = 229.

Stepwise generalized linear equation, *n* = 148.

Not significant: maternal diabetes mellitus, medical insurance (Medicaid vs other or none), small for gestational age, sex.

^a^Pre-pregnancy or first trimester.

*BMI* body mass index.

### Subsequent serum Zn levels

The lowest serum Zn level was lower in Epoch-1 than in Epoch-2 (Table 2a); however, in Epoch-1 levels were only obtained in those with growth failure. In Epoch-2, ELGANs had a median of 20 samples, (1.25,3.0) while those 29-33wks GA had 1.0 (1.0, 2.0). Among 104 ELGANS, 28 had 1 sample [median length of stay (LOS) 75 days], 44 had 2 samples (LOS 71 days, 23 had 3 samples (LOS 100 days) and 11 had 4 samples (LOS 102 days).

**Fig. 3:**
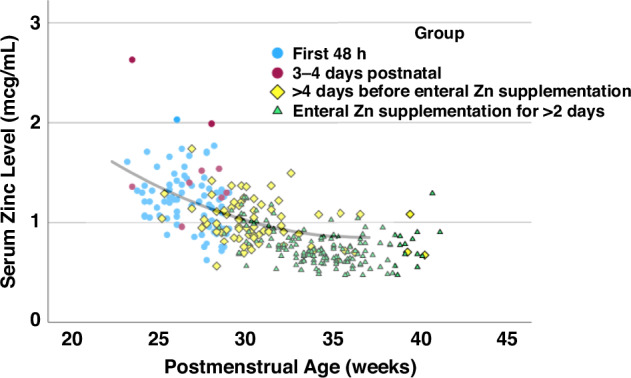
Serum zinc level versus postmenstrual age among infants < 29 weeks gestational age in Epoch-2 in the growth cohort. Blue circles represent serum zinc levels within the first 48 h postnatal while red circles represent levels at 3–4 days of life. Yellow diamonds show zinc levels at > 4 days of life before enteral zinc supplementation and green triangles represent zinc levels after >2 days of enteral zinc supplementation. The line shows the estimate from the quadratic regression analysis based on serum Zn levels within the 48 h postnatal among all infants (Fig. 2). Yellow diamonds are evenly distributed around the line, whereas many green triangles after 32 weeks postmenstrual age are below the line. Zn serum zinc level, GA gestational age, PMA postmenstrual age.

In Epoch-2, serum Zn levels decreased with increasing GA and PNA and with feeds and enteral Zn supplementation (*P* < 0.001) (Supplementary Table 2). Serum Zn levels by GA stratum decreased with PMA (Supplementary Figure 1). Among ELGANs, serum Zn levels during the first week of life and those on PN were similar to initial levels when plotted against PMA. In contrast, many levels obtained after 32wks PMA with enteral Zn supplementation for at least 2 days were lower than expected (Fig. 3). Serum Zn levels did not change significantly after increasing the routine dose of enteral Zn (Table 5).

**Table 5:** Serum zinc levels by gestational age and postnatal age in Epoch-2 in the growth cohort. a. Serum zinc levels. b. Frequency of serum zinc levels < 0.74 mcg/mL.

a					
GA (weeks)		Postnatal age (days)			
	Total N of samples	0–1 ( ≤ 48 h)	2–20	21–41	≥ 42
22–28	320	1.21 (0.98, 1.37) (83)^a^	1.03 (0.88, 1.27) (53)^b^	0.94 (0.77, 1.09) (57)^c^	0.69 (0.61, 0.81) (127)^c^
29–33	436	0.89 (0.79, 1.03) (143)^a^	0.83 (0.73, 0.94) (167)^b^	0.73 (0.64, 0.85) (76)^c^	0.76 (0.66, 0.86) (50)^c^

b						
GA (weeks)	N/total individuals with any serum zinc level < 0.74 mcg/ml in NICU*		N/total Samples with serum zinc level < 0.74 mcg/mL at Various postnatal ages (days)**			
		Total N of samples	0–1 ( ≤ 48 h)	2–20	21–41	≥ 42
22–28	69/111 (62.2%)	320	2/83 (2%)^a^	3/53 (6%)^a b^	10/57 (18%)^b^	79/127 (62%)^c^
29–33	105/228 (46.1%)	436	18/143 (12%)^a^	42/167 (25%)^b^	40/75 (53%)^c^	21/50 (42%)^bc^

Values are median (interquartile range) (n).

Three-way ANOVA by rank, *n* = 755, gestational age *P* < 0.001, postnatal age *P* = 0.001; Date of birth up to vs after 6/30/2022* *P* = 0.66.

Different alphabetic superscripts across columns indicate significant pair-wise differences.

*The routine total dose of zinc enteral intake was increased from 2.5 mg/kg/day to 2.7–3 mg/kg/day on 7/1/22 as recommended by Koletzko et al. 2022.

Values are number/total (%).

*Chi-square analysis across rows.

** Fisher’s exact test across columns followed by pair-wise comparisons using Bonferroni correction, *P* < 0.001.

Different alphabetic superscripts across columns indicate significant pair-wise differences.

*NICU* neonatal intensive care unit.

The frequency of low serum Zn levels by GA stratum increased with PNA (Table 5.b). At least one serum Zn level was low in 62% among ELGANs and 46% among 29–33 wks GA infants. Among 172 infants with a low serum Zn level, 61 (35.4%) had at least one repeat level. Among 31 with a low repeat level, all had the dose of enteral Zn increased, while among 30 with a normal repeat level, 24 (80%) had the dose increased.

There was a larger percentage of infants with growth failure that had concomitant Zn deficiency in Epoch-2 vs Epoch-1 (42.1% vs 8.2%, *P* < 0.001). A larger percentage of infants with growth failure had either low serum Zn or low vitamin D level or both, in Epoch-2 vs Epoch-1 (68.4% vs 29.2%, *P* < 0.001) (Supplementary Table 3).

### Morbidity and mortality

Morbidity and mortality outcomes were similar among 530 infants in Epoch-1 and 384 infants in Epoch-2 (Fig. 1), except for a shift from severe to moderate BPD from Epoch-1 to Epoch-2, respectively (*P* = 0.03) (Table 6).

**Table 6:** Morbidities and mortality in Epochs 1 and 2 among all infants excluding those on comfort care only.

Variable	Epoch-1 *N* = 530	Epoch-2 *N* = 384	*P*-value
< 33 weeks GA or < 1500 grams birthweight			
Severe congenital anomaly	20 (3.8%)	16 (4.2%)	0.76
Duration of mechanical ventilation (days)	3 (1,15)	3 (1,14)	0.26
Duration of CPAP (days)	28 (6,44)	30 (5,45)	0.27
BPD stage (NIH consensus definition)			0.03
None	455 (85.8%)	322 (83.9%)	
Mild	28 (5.3%)	22 (5.7%)	
Moderate	10 (1.9%) ^a^	20 (5.2%) ^b^	
Severe	37 (7.0%)	20 (5.2%)	
% infants with >10% glucose values > 150 mg/dL			
22–28 weeks GA	38/146 (26%)	29/103 (28%)	0.71
29 weeks GA	5/333 (2%)	6/235 (3%)	0.54
% of glucose values > 150 mg/dL per patient			
22–28 weeks GA	1.4 (0.0,10.6)	2.1 (0.0,11.3)	0.14
29 weeks GA	0 (0,0)	0 (0,0)	0.65
Late onset sepsis	29 (5.5%)	27 (7.1%)	0.33
NEC stage II	22 (4.2%)	19 (4.9%)	0.57
NEC stage III	13 (2.5%)	5 (1.3%)	0.24
Severe IVH grade (III or IV)	23 (4.3%)	23 (6.0%)	0.26
Severe ROP (Avastin or laser)	29 (5.5%)	18 (4.7%)	0.60
Mortality to discharge	39 (7.4%)	27 (7.0%)	0.85
Only < 33 weeks GA			
Any ROP	130/502 (25.9%)	86/360 (23.9%)	0.50
Only < 29 weeks GA			
Severe ROP (Avastin or laser)	29/177 (16.4%)	18/122 (14.8%)	0.70

Values are median (interquartile range) or number (%).

Mann-Whitney test or Chi-square analysis followed by pair-wise comparisons with Bonferroni correction.

Different alphabetic superscripts across columns indicate significant pair-wise differences.

*GA* gestational age, *CPAP* continuous positive airway pressure, *BPD* bronchopulmonary dysplasia, *NECx* necrotizing enterocolitis, *IVH* intraventricular hemorrhage, *ROP* retinopathy of prematurity.

In Epoch-2, morbidities were compared in 3 groups by serum Zn level (Supplementary Table 7). Since GA was lower among those with high serum levels, morbidity was analyzed in two GA strata. There was no increased morbidity or mortality among infants with low or those with high serum Zn levels compared with those with normal levels.

GA-adjusted odds of mortality, sepsis, severe BPD, NEC and severe IVH were not higher among infants with any serum Zn level >1.46 mcg/mL or among infants with any level >95% CI of the quadratic equation versus infants with all levels within the respective limit (Supplementary Table 4).

### ROP in infants born at < 33 weeks GA

Since all infants who developed ROP were born at < 33wks GA, analyses were limited to 502 < 33 week-GA infants in Epoch-1 and 360 in Epoch-2 (Fig. 1). In bivariate comparisons in both epochs ROP was associated with lower GA, lower BW, any growth failure, higher rate of weight gain to discharge, respiratory distress syndrome (RDS), surfactant administration, worse BPD, and documented sepsis (Supplementary Table 5.1). In Epoch-2, ROP was also associated with lower minimum serum Zn level and low serum Zn and vitamin D level (Supplementary Table 5.2). ROP was also associated with high initial serum Zn level but normal ratio of observed to expected serum Zn level for GA. Furthermore, nonparametric ANCOVA of initial Zn level and GA showed no difference among infants with versus those without ROP (*P* = 0.139).

In multivariable analysis in both epochs, ROP was associated with low GA and BW, higher stage of BPD, and growth failure pattern (Supplementary Table 5.3). This first model had an AUC value of 0.867 (95% CI 0.842, 0.892) and poor fit with the data (Hosmer-Lemeshow test, *P* = 0.002).

In multivariable analysis in Epoch-2 ROP was associated with low GA and BW, higher stage of BPD and lowest serum Zn level (Supplementary Table 5.4). This second model had an AUC of 0.928 (95% CI 0.899, 0.957), was internally validated, and had better fitting than the first model when the latter was applied to Epoch-2 only (Supplementary Figure 2).

### Severe ROP in ELGANs

Since all infants who developed severe ROP were ELGANs, analyses were limited to 122 ELGANs in Epoch-2. In bivariate analysis, severity of ROP was associated with low GA, low BW, low minimum serum Zn level and both low serum Zn and low vitamin D level (Supplementary Table 5.5). In multivariable analysis adjusted for GA, BW and stage of BPD, both mild/moderate and severe ROP were associated with any low serum level of Zn or 25-hydroxyvitamin D (Supplementary Table 5.6). This model was internally validated. If used to predict any ROP, this model has a sensitivity of 83.6% and specificity 67.3%. Among 18 infants with severe ROP, this model would classify 17 infants as having ROP (sensitivity 94.4%).

### Validation of results from the NICU database

Validation of data extracted from the NICU database among infants born in 2022-2023 demonstrated an intraclass correlation coefficient close to 1 for GA and BW and Cohen kappa of at least 0.976 for NEC, SIP, and severe IVH in ELGANs (Supplementary Table 6).

## Discussion

### Zn and growth parameters

In this study, the initiation of routine enteral Zn supplementation to meet daily recommendations and routine serial serum Zn measurements resulted in 20% improvement in adjusted Δlength_z_ among infants in Epoch-2 in comparison to Epoch-1, in the absence of any significant change in energy or protein intake. There was no significant impact on Δweight_z_ or ΔFOC_z_. More infants in Epoch-2 experienced physiologic growth patterns. Overall, the findings suggest that routine enteral Zn supplementation along with routine serial serum Zn measurements improves linear growth and overall growth pattern in VPT/VLBW infants.

Improved growth has been one of the more consistent findings in RCTs of Zn supplementation that included preterm newborns. Most studies show an improved effect on linear growth and weight, but not consistently on FOC.^19,41^ In contrast, in a pre and post study which included 221 newborns, Ogasawara et al reported no change in anthropometric measurements at discharge after implementing routine enteral Zn supplementation (3 mg/kg/day) starting at 2 weeks of life to discharge without PN Zn.^42^ Their primary outcome was the proportion of infants greater than median weight, length and FOC at discharge; however, no information about methods of anthropometric measurements was provided. In our study, which included overall less mature newborns, Zn was provided both enterally and in PN; in Epoch-2, intravenous Zn dosage was increased in addition to routine enteral Zn supplementation, and Zn dosage was adjusted if serum Zn levels were low. In addition, although Ogasawara et al observed an increase in serum Zn levels after implementing Zn supplementation, Zn levels at discharge were lower when compared to our study (0.65 vs 0.77 mcg/mL in Epoch-2). In another study, improvement in linear growth was reported with higher enteral doses of Zn (10 mg/day) in VLBW.^43^ Our group recently demonstrated that in Zn-deficient ELGANs, low dose Zn supplementation for at least 2wks improved FOC, but not linear growth and weight.^44^ Variations in Zn dosing (enteral and IV), duration of treatment and differences in primary outcomes of each study, need to be carefully assessed when conclusions are made about the efficacy of Zn provision. Further studies are needed to delineate the best dosing and duration of Zn supplementation in VPT/VLBW infants.

### Initial serum Zn level

We describe a quadratic fit for the decrease of the initial level with GA (Fig. 2). These changes agree with previous studies.^42,45^ The inverse relation in Zn levels with GA is not well understood. Presence of high Zn turnover with decreasing GA, volume expansion with increasing GA, lower tissue utilization of Zn with lower GA and increased Zn clearance from the kidneys with increasing GA, could all contribute to the presence of higher serum Zn levels earlier in gestation.^46,47^ In addition, maternal levels decrease with gestation and are positively correlated with cord and early neonatal levels.^48,49^ Since circulating Zn represents ~0.1% of the total body content, the role of serum levels needs to be carefully interpreted. Zn is stored in specific proteins (Metallothioneins), especially in the hepatocyte and can be released upon net deficit and redox changes.^45,50,51^ In circulation, Zn is transferred in the red blood cell, and in serum, bound to albumin and other proteins, while interaction at the cellular level occurs via specialized receptors.^52^ In Epoch-2 the initial Zn level was low in up to 12% of newborns between 29 and 33wks GA, thereby suggesting possible maternal and fetal Zn deficit. Lower Zn intake is encountered frequently in mothers with low poverty index and in women of Mexican origin.^53^ Maternal Zn deficiency could be more prevalent at PHHS which cares for a high proportion of underprivileged, lower income maternal patients.

### Subsequent Zn levels

Among ELGANs on PN, the subsequent decrease in Zn levels with PNA and PMA matched the decrease in serum Zn as expected from the quadratic regression line, suggesting that PN Zn intake was sufficient to meet intrauterine accretion (Supplementary Figure 1). In contrast, the lower serum levels among ELGANs with enteral feeding despite enteral Zn supplementation vs expected levels after 32wks of PMA (Supplementary Figure 1) and the increasing frequency of low serum Zn levels with increasing PNA in both GA groups in Epoch-2 suggest that enteral Zn intake per current guidelines was insufficient. The higher frequency of Zn deficiency diagnosis in Epoch-2 than in Epoch-1 is likely an artifact resulting from the fact that serum Zn levels were only collected among infants with poor growth in Epoch-1.

If data from this study are confirmed in other studies, the normative range for serum Zn levels in preterm infants born in countries without endemic Zn deficiency may need to be reconsidered. First, data from this study and from Terrin^45^ suggest that levels decrease with PMA and PNA. Second, no toxicity was observed in this study despite serum Zn levels >1.46 mcg/mL. Previous data^44^ supports a value of 0.74 mcg/mL as lower limit of normal at 30-45wks PMA.

The total enteral intake of Zn (2-3 mg/kg/day), based on current recommendations, does not match transplacental fetal Zn accretion and as result actual needs for Zn might not be met.^45,54^ The low Zn levels could reflect a total body Zn deficit that may be due to a delayed ability of the preterm infant’s GI tract to absorb Zn because of a decrease in Zn-binding ligands or depleted Zn stores due to increased nutritional requirements.^55–58^ Factors that could contribute to this finding include endogenous losses, decreased ability to absorb Zn, and dependency on DHM.

The absorption of Zn in the gut occurs with Zn-irk like receptors (ZIP), with competition with other micronutrients (sodium, Cu, magnesium, iron and calcium)^52^ and possibly facilitated by an intact gut microbiome.^59,60^ Absorption of Zn occurs in the brush border of intestinal mucosa especially in the duodenum and jejunum.^61^ Differentiation of the enterocyte border and effective length of the jejunum increases with GA.^62^ For the above reasons, bioavailability of enteral Zn in preterm newborns is only 10%–30%; therefore, enteral Zn needs in preterm infants are estimated as minimum of 4–5 mg/kg/day to match fetal intake.^45,63–65^ A study with radiolabeled Zn in preterm newborns (mean GA 31wks), showed a positive correlation of increased daily weight gain and intestinal Zn absorption when Zn was supplemented at 3 mg/kg/day, which matches what was provided in our study.^66^ Fortification with human milk fortifiers and preterm formula provides about 2 mg/kg Zn per day.^67^ The level of Zn in MOM is twice that in maternal serum.^68^ The total quantity of Zn transferred to the newborn exclusively by MOM can reach up to 0.5–1.0 mg per day.^69^ Zn content in MOM after term delivery decreases postpartum from a content of 8-12 mg/L in colostrum to 0.7 to 1.6 mg/L by 1 month.^54,70^ Zn content in DHM, as measured in 11 DHM samples and reported from our group, was 2.14 ± 0.73 mg/L.^23^ In contrast, preterm MOM has higher Zn content of 2-9 mg/L until 4-12wks postnatal age.^71,72^ In our cohort, in both epochs, DHM was used as a primary alternative, when MOM was not available. Formula was avoided until 36wks PMA, unless mother declined DHM.

### Zn levels and long-term neurodevelopmental outcomes

Postnatal growth (assessed by FOC, length, weight, growth velocity, growth pattern, and/or body mass index, BMI) could be independently associated with improved neurodevelopmental outcomes in preterm infants.^73–76^ Zn supplementation, on the other hand, could impact growth positively, but its role in neurodevelopment is not well studied. So far only three RCTs of Zn supplementation reported early, up to 1-year in follow up, neurodevelopmental outcomes in preterm neonates.^77–79^ In addition, whether low Zn levels are associated with abnormal neurodevelopmental outcomes is poorly investigated. Terrin et al. showed that in preterm newborns 23-34wks GA, there was a positive correlation between total composite motor scores (with Bayley III scale) and serum Zn levels at 28 days of life (DOL) (R = 0.467, *P* < 0.05).^80^ The same authors found that serum Zn levels at 28 DOL were associated with energy and protein intake (*P* < 0.001) through PN in the first week of life. The association of Zn levels, nutrient intake and neurodevelopment needs further research. The long-term neurodevelopmental outcomes of very preterm infants included in the current study will be carefully assessed.

### Zn and ROP

In this study, ROP was associated with decreasing GA, lower BW, lowest serum Zn level, and severity of BPD. ROP develops in two phases: (1) a hypoxic phase and (2) a proliferative phase. Phase 1 has low insulin-like growth factor (IGF-1) and VEGF and is associated with slow postnatal growth while phase 2 has increasing IGF-1 production with high VEGF.^81^ The link of ROP with Zn deficiency is supported by physiologic data and a few observational studies.

The retina and choroid contain the highest Zn among any other organ (464–472 mcg/g Zn versus 20–30 mcg/g of dry weight, respectively).^82^ Zn acts as an inhibitory signal to glutaminergic transmission and as a protective signal against excitotoxicity.^83,84^ Glutamate toxicity can induce VEGF, a key molecule for development of ROP.^85^ Early Zn supplementation in preterm infants with RDS increases the activity of superoxide dismutase, a key antioxidant enzymatic system.^86^ Preclinical studies show that retinal cells use Zn at high levels to prevent oxidative stress and toxicity from physiologic signals. We would expect that Zn deficiency to be associated mechanistically with ROP.

Several observational studies have been conducted to investigate the association of Zn with ROP. In a case-control study preterm newborns with ROP had lower maternal, cord and serum Zn levels at 40wks PMA when compared to those who did not develop ROP.^87^ In another study, Yang et al. showed that Zn levels were higher within a few days after birth among infants with ROP versus term infants;^88^ a finding which likely resulted from the negative relationship between serum Zn levels and GA (as shown in the current study). Mishra et al., in a retrospective study including infants 28–37wks GA, reported a higher frequency of ROP in newborns with Zn levels <0.7 mcg/mL <24 h postnatal than in those with higher Zn level, both in bivariate (42% vs 24% respectively, *P* = 0.02) and multivariable analysis (adjusted OR = 2.22).^89^ Meta-analyses of RCTs that included infants with BW < 2.5 kg and GA <37wks found that enteral Zn supplementation compared to no supplementation or placebo had no effect on ROP.^19,41^ However, these RCTs included infants >32wks GA, with low baseline risk of ROP, and used lower (and short) Zn supplementation than currently recommended.

### Zn, Vitamin D and possible association with ROP, inflammation, and other outcomes

Our second predictive model for ROP including serum Zn level has a better AUC (0.928) (Supplementary Figure 2) than models using BW, GA with or without postnatal weight gain (AUC 0.726-0.901).^90–92^ but similar to artificial intelligence models using retinal imaging (AUC 0.91–0.99).^81,93^.

Vitamin D integrity and functions are tightly regulated by Zn.^94,95^ Zn supplementation increases serum vitamin D levels and there is a positive correlation between serum vitamin D levels and Zn levels in children and adults.^96–98^ In one study preterm newborns with Stage 1 ROP had lower tear levels of vitamin D and VEGF while those with Stage 3 ROP had higher tear vitamin D levels with low VEGF.^99^ It is proposed that Stage 1 ROP resembles a hyperoxic condition, where vitamin D would have pro-angiogenic (or pro-VEGF) activity, whereas Stage 3 ROP mimics a hypoxic environment, in which vitamin D would have anti-angiogenic (or anti-VEGF) activity.^99^

### Strengths and limitations

Strengths of the study include large sample size; gold standard method for serum Zn and vitamin D measurements; accurate serial length measurements and multivariable analyses allowing to adjust for confounding variables; and strong performance of predictive models for ROP. To our knowledge this is the largest study to date to describe the postnatal trajectory of serum Zn levels while receiving currently recommended Zn supplementation.

Limitations of the study include possible unrecognized bias due to a single-center pre/post cohort study, inability to assess cause-and-effect relationships, limited generalizability due to a specific racial/ethnic mix and high percentage of Medicaid coverage, missing samples due to hemolysis, lack of maternal serum Zn level, limitation of serum Zn measurements in Epoch-1 to those with growth failure and limitation of serum 25-OH vitamin D measurements in both epochs to those with growth failure, and increased routine enteral Zn supplementation in the middle of Epoch-2. Logistic models for ROP were developed post-hoc and hence need validation from further well-designed studies. Multiple secondary analyses were done without adjusting *P* values and without sample size analysis; therefore, these analyses should be considered exploratory.

### In conclusion

In this study, implementing a protocol that includes increased PN Zn and routine enteral Zn prophylaxis to meet current recommendations and serial serum Zn levels to prevent and/or treat Zn deficiency earlier in VPT/VLBW infants was associated with improvement in linear growth and growth patterns but not morbidity and mortality. There was no evidence of toxicity from Zn supplementation even with transient high serum Zn levels. Since low serum Zn levels were frequent during enteral supplementation, it is possible that currently recommended dose (up to 3.5 mg/kg/day) is not sufficient to optimize outcomes. We have developed and validated a predictive model of ROP using GA, BW, stage of BPD and the lowest serum Zn level. Assessing the initial serum Zn level could be indicative of fetal Zn deficiency. The ratio of initial Zn level to expected level for GA as described by the quadratic equation might be a promising marker of early Zn assessment but needs further validation.

Since Zn deficiency is asymptomatic in most cases and common as shown here, monitoring of Zn levels could be beneficial. Multicenter studies are needed to assess whether dosage of enteral zinc should be increased and whether zinc could help prevent ROP.

## Supplementary information

Supplementary Material

## Data Availability

The datasets generated and/or analyzed during the current study are available from the corresponding author on reasonable request.
