# Supplementary material for: Improved linear growth after routine zinc supplementation in preterm very low birth weight infants
Source: Pediatr Res. 2025 Mar 11;98(4):1436–48. doi: 10.1038/s41390-025-03935-z (PMC12549326; doi:10.1038/s41390-025-03935-z)

Supplementary Material

Supplementary Table 1. Comparison of baseline maternal and neonatal characteristics among infants in the Growth Cohort vs those with congenital anomaly or death in the NICU

Supplementary Table 2. Multivariate analysis of ranks of serum zinc levels by gestational age, postnatal age and enteral zinc supplementation prior to blood sampling in Epoch-2 in the growth cohort

Supplementary Table 3. Serum vitamin D and Zn Levels vs Growth Failure in both epochs the growth cohort

Supplementary Table 4. Assessment of toxicity versus high serum zinc levels among all infants excluding those on comfort care only in Epoch-2

Supplementary Table 5. Association of ROP with other variables

Supplementary Table 5.1. Bivariate analyses in both epochs among all infants < 33 weeks excluding comfort care only

Supplementary Table 5.2. Bivariate analyses in Epoch-2 among all infants < 33 weeks excluding comfort care only

Supplementary Table 5.3. Multivariate analysis of ROP in both epochs among infants < 33 weeks excluding comfort care only, n=862

Supplementary Table 5.4. Multivariate analysis of ROP in Epoch-2 among infants < 33 weeks excluding comfort care only, n=329

Supplementary Table 5.5. Lowest serum zinc level vs severity of ROP in Epoch-2 among infants <29 weeks gestational age excluding comfort care only

Supplementary Table 5.6. Internal validation of model of retinopathy of prematurity vs any low serum level of zinc or vitamin D in Epoch-2 among infants < 29 weeks excluding comfort care only, n=122

Supplementary Table 6. Validation of results extracted from the NICU database among infants born in 2022-2023

Supplementary Table 7. Morbidities and mortality in Epoch-2 versus zinc level among infants born at < 33 weeks of gestational age,

Supplementary Figure 1. Serial serum zinc levels vs postmenstrual age and postnatal age by gestational age group in the growth cohort in Epoch-2

Supplementary Figure 2. Receiver operating curves and calibration curves for the two models predicting ROP in 329 preterm infants < 33 weeks gestational age in Epoch-2, excluding those with comfort care only

Supplementary Table 1. Comparison of baseline maternal and neonatal characteristics among infants in the growth cohort vs those with congenital anomaly or death in the NICU, excluding comfort care only

Characteristics	Infants in the Growth Cohort	Infants with Congenital Anomalies or Death in NICU	*P*-value*
**Maternal**	**N=775**	**N=31**	
Prenatal/first trimester maternal BMI (kg/m^2)	31.94±7.52 (592)	31.40±6.43 (23)	0.74
Multiple gestation	101 (13.0%)	4 (12.9%)	*1.00
Prenatal care	725 (93.5%)	31 (100%)	0.25
Pregnancy-induced hypertension	332 (42.8%)	12 (38.7%)	0.71
Diabetes mellitus	127 (16.4%)	3 (9.7%)	*0.46
Antenatal steroids	681 (87.9%)	24 (77.4%)	0.10
Magnesium	437 (56.4%)	10 (32.3%)	0.05
Race/Ethnicity	N=734	N=31	*0.49
Hispanic	498 (67.8%)	24 (77.4%)	
Non-Hispanic Black	195 (26.6%)	5 (16.1%)	
Non-Hispanic White	30 (4.1%)	1 (3.2%)	
Other	11 (1.5%)	1 (3.2%)	
Cesarean Delivery	505 (65.2%)	23 (74.2%)	0.34
**Neonatal**	**N=878**	**N=36**	
Female	433 (49.3%)	21 (58.3%)	0.29
Gestational age (weeks)	30 (27, 32)	32 (29, 32)	0.01
Birthweight (grams)	1400 (1020, 1690)	1445 (1001, 1825)	0.60
Weight Z-score	0.06±1.21	-0.38±1.32	0.03
Apgar score at 5 minutes	8 (6,9)	7 (4,8)	0.03
Admission temperature (degrees centigrade)	36.2±0.7	36.0±0.7	0.08
Admission temperature			*0.36
36.5-37.5^{0}C	253 (29.0%)	8 (23.5%)	
<36.5^{0}C	588 (67.4%)	26 (76.5%)	
>37.5^{0}C	31 (3.6%)	0 (0%)	
Small for gestational age	117 (13.3%)	8 (22.2%)	0.13
Birth FOC (cm)	27.5 (25.4, 29.0)	27.3 (25.4, 29.50)	0.92
FOC Z-score	-0.09±1.12	-0.59±1.31	0.01
Accurate length in 1st week (cm)	38.7 (35.6, 40.9) (613)	40.3 (37.5, 42.4) (13)	0.09
Accurate length Z-score	-0.29±1.62	-0.38±1.51	0.83
BMI using first accurate length (kg/m^2)	8.39 (7.57, 8.16)	8.82 (8.16, 9.69)	0.10
BMI Z-score	-0.43±1.84	-0.37±1.67	0.86

Values are mean±SD (n), median (interquartile range) or number (%).
Student t-test, Mann-Whitney test, chi-square analysis or *Fisher's exact test.
Different alphabetic superscripts across columns indicate significant pair-wise differences.
Abbreviations: NICU, neonatal intensive care unit; BMI, body mass index; FOC, fronto-occipital circumference

Supplementary Table 2. Multivariate analysis of ranks of serum zinc levels by gestational age, postnatal age and enteral zinc supplementation prior to blood sampling in Epoch-2 in the growth cohort

**Variable**	**Beta Coefficient (95% CI)**	***P*-Value**
Gestational age (weeks)	-25 (-31, -18)	<0.001
Postnatal age (days)	-3.0 (-4.4, -1.6)	<0.001
Routine enteral zinc supplementation for > 2 days	-139 (-191, -86)	<0.001

Mixed model generalized linear equation by rank, 768 measurements among 352 neonates

Supplementary Table 3. Serum Vitamin D and Zinc Levels vs Growth Failure by epoch in the growth cohort

Serum zinc and vitamin D levels	Epoch-1			Epoch-2			P-value Epoch-1 vs Epoch-2
	No GF	GF	*P*-value	No GF	GF	*P*-value	
Neither measured	238 (77.0%) [a]	89 (52.0%) [b]	<0.001*	16 (5.9%)	2 (2.6%)	<0.001*	<0.001 *
Both or either normal	14 (4.5%) [a]	32 (18.7% [b]		116 (43.0%) [a]	22 (28.9%) [b]		
Low serum zinc level	37 (12.0 %)	14 (8.2%)		112 (41.5%)	32 (42.1%)		
Low serum vitamin D level	19 (6.1%) [a]	29 (17.0%) [b]		12 (4.4%)	4 (5.3%)		
Both low	1 (0.3%) [a]	7 (4.1%) [b]		14 (5.2%) [a]	16 (21.1%) [b]		
Total	309	171		270	76		
Either zinc or vitamin D level low or both low	57 (18.4%)	50 (29.2%)	0.007**	138 (51.1%)	52 (68.4%)	0.007 **	<0.001 **

Values are number (%).
Multinomial or binary logistic regression analysis followed by *Fisher's exact test or ** chi-square analysis
Different alphabetic superscripts across columns indicate significant pair-wise differences.
Abbreviations: GF: growth failure affecting weight or length or both
Low serum zinc level: <0.74 mcg/mL; low vitamin D level: <30 mcg/mL

Supplementary Table 4. Assessment of toxicity versus high serum zinc levels among all infants in Epoch-2 excluding those on comfort care only

Variable	Adjusted OR (95% CI) in 26 infants with any serum zinc level >1.46 mcg/mL vs 358 with all levels ≤1.46 mcg/mL	Adjusted OR (95% CI) in 29 infants with any serum zinc level >95% CI of quadratic regression vs 355 with all levels within that limit
Mortality (n=27)	0.468 (0.099, 2.224)	*
Late onset sepsis (n=27)	2.181 (0.733, 6.488)	0.500 (0.106, 2.365)
Severe bronchopulmonary dysplasia (n=20)	1.411 (0.372, 5.358)	1.222 (0.286, 5.213)
Necrotizing enterocolitis (n=19)	0.470 (0.057, 3.840)	*
Severe intraventricular hemorrhage (n=23)	2.680 (0.875, 8.211)	2.298 (0.723, 7.305)

Logistic regression analysis adjusted for gestational age
Abbreviations: OR, odds ratio; CI, confidence interval
*Cannot be estimated (no cases among infants with high serum Zn level

Supplementary Table 5. Association of ROP with other variables

Supplementary Table 5.1 Bivariate analyses in both epochs among all infants < 33 weeks excluding comfort care only

Variable	N	No ROP N=646	ROP N=216	*P* value	OR (95% CI)	*P* value
Epoch-2 [N=360] (vs Epoch-1 [N=502])	862	274 (42.4%)	86 (39.8%)	0.50	0.898 (0.656, 1.229)	0.50
Gestational age (weeks)	862	31.1 (29.4, 32.1)	27.4 (25.1, 28.6)	<0.001	0.604 (0.561, 0.650)	<0.001
Birthweight (grams)	862	1550 (1224, 1790)	920 (750, 1198)	<0.001	0.997 (0.996, 0.997)	<0.001
Initial weight loss > expected[1]	862	142 (22.0%)	51 (23.6%)	0.62	1.097 (0.762, 1.580)	0.62
Any growth failure pattern in NICU	862	132 (20.4%)	116 (53.7%)	<0.001	4.517 (3.251, 6.275)	<0.001
Rate of weight gain (g/kg/day)[2]	836	10.2 (8.4, 12.0)	12.2 (11.0, 13.3)	<0.001	1.298 (1.210, 1.393)	<0.001
Stage of BPD (NIH consensus)	862			*<0.001		<0.001
None		620 (96.0%) [a]	105 (48.6%) [b]		Ref	-
Mild		14 (2.2%) [a]	36 (16.7%) [b]		15.184 (7.918, 29.116)	<0.001
Moderate		4 (0.6%) [a]	26 (12.0%) [b]		38.381 (13.128, 112.208)	<0.001
Severe		8 (1.2%) [a]	49 (22.7%) [b]		36.167 (16.654, 78.540)	<0.001
Maximum respiratory support	862			*<0.001	5.493 (3.923, 7.691)**	<0.001
None or nasal cannula		69 (10.7%) [a]	0 (0%) [b]		-	
CPAP or NIPPV		498 (77.1%) [a]	122 (56.5%) [b]		-	
Invasive ventilation		79 (12.2%) [a]	94 (43.5%) [b]		-	
Surfactant	862	249 (38.5%)	158 (73.1%)	<0.001	4.343 (3.091, 6.103)	<0.001
Respiratory distress syndrome	862	511 (79.1%)	215 (99.5%)	<0.001	56.80 (7.892, 408.79)	<0.001
Documented sepsis	862	30 (4.6%)	26 (12.0%)	<0.001	2.810 (1.621, 4.869)	<0.001

Values are mean±SD, median (interquartile range) or number (%).

Student t-test, Mann-Whitney test, chi-square analysis, *Fisher's exact test or unadjusted logistic regression analysis

** vs none (subgroups cannot be assessed because of zero in first group of ROP)

Different alphabetic superscripts across columns indicate significant pair-wise differences.

Abbreviations: ROP, retinopathy of prematurity; OR, odds ratio; CI, confidence interval; NICU, neonatal intensive care unit; BPD, bronchopulmonary dysplasia by NIH consensus classification (Jobe); CPAP, continuous positive airway pressure; NIPPV, nasal intermittent positive pressure ventilation

[1] during the first 4 weeks postnatal, compared with Rochow's curve

[2] from birth to discharge using Patel geometric method

There was no difference in race/ethnicity or sex between the two groups.

Supplementary Table 5.2 Bivariate analyses in Epoch-2 among all infants < 33 weeks excluding comfort care only

Variable	N	No ROP N=274	ROP N=86	*P* value	OR (95% CI)	*P* value
Gestational age (weeks)	360	31.1 (29.6, 32.1)	26.4 (25.0, 28.4)	<0.001	0.579 (0.514, 0.651)	<0.001
Birthweight (grams)	360	1567 (1238, 1793)	821 (709, 1061)	<0.001	0.996 (0.995, 0.997)	<0.001
Growth failure pattern	357			<0.001		<0.001
None		237 (87.5%) [a]	45 (52.3%) [b]		Ref	-
Yes, no low serum zinc level		16 (5.9%)	10 (11.6%)		3.292 (1.404, 7.716)	0.006
Yes, low serum zinc level		18 (6.6%) [a]	31 (36.0%) [b]		9.070 (4.676, 17.593)	<0.001
Rate of weight gain (g/kg/day)[1]	345	10.2 (8.5, 12.0)	12.4 (11.4, 13.4)	<0.001	1.316 (1.177, 1.472)	<0.001
First serum zinc level (mcg/mL) during the first 48 hours	224	0.90 (0.79, 1.05) (n=161)	1.17 (0.95, 1.37) (n=63)	<0.001	25.008 (8.927, 70.054)	<0.001
Ratio of observed/expected zinc level during the first 48 hours [2]	224	0.99±0.21 (n=161)	1.03±0.23 (n=63)	0.18	2.523 (0.651, 9.773)	0.18
Lowest serum zinc level (mcg/mL)	329	0.75 (0.67,0.86)	0.66 (0.60, 0.76)	<0.001	0.059 (0.011, 0.307)	<0.001
Serum zinc level < 0.74 mcg/mL	329			<0.001		<0.001
None		133 (54.7%) [a]	27 (31.4%) [b]		Ref	-
Starts at ≥34 weeks PMA		71 (29.2%)	31 (36.0%)		2.151 (1.191, 3.883)	0.01
Starts <34 weeks PMA		39 (16.0%) [a]	28 (32.6%) [b]		3.537 (1.869, 6.693)	<0.001
Serum zinc and vitamin D level[3]	360			*<0.001	2.053 (1.596, 2.641)**	<0.001
Neither measured		30 (10.9%) [a]	0 (0%) [b]			
Both or either normal		123 (44.9%) [a]	20 (23.3%) [b]			
Low serum zinc level		95 (34.7%) [a]	43 (50.0%) [b]			
Low serum vitamin D level		11 (4.0%)	7 (8.1%)			
Both low		15 (5.5%) [a]	16 (18.6%) [b]			
Stage of bronchopulmonary dysplasia[4]	360			*<0.001		<0.001
None		262 (95.6%) [a]	36 (41.9%) [b]		Ref	-
Mild		7 (2.6%) [a]	15 (17.4%) [b]		15.595 (5.957, 40.829)	<0.001
Moderate		3 (1.1%) [a]	17 (19.8%) [b]		41.241 (11.514,147.72)	<0.001
Severe		2 (0.7%) [a]	18 (20.9%) [b]		65.500 (14.588, 249.09)	<0.001
Maximum respiratory support	360			<0.001	7.656 (4.385, 13.366)**	<0.001
None or nasal cannula		29 (10.6%)	0 (0%)			
CPAP or NIPPV		218 (79.6%)	46 (53.5%)			
Invasive ventilation		27 (9.9%)	40 (46.5%)			
Surfactant	360	108 (39.4%)	69 (79.1%)	<0.001	5.807 (3.273, 10.301)	<0.001
Respiratory distress syndrome	360	232 (84.7%)	86 (100%)	<0.001	5.807 (3.273, 10.301)	<0.001
Documented sepsis	360	15 (5.5%)	12 (14.0%)	0.01	2.800 (1.256, 6.243)	0.01

Values are mean±SD, median (interquartile range) or number (%).
Student t-test, chi-square analysis, *Fisher's exact test or unadjusted logistic regression analysis
** vs none (subgroups cannot be assessed because of zero in first group of ROP)
Different alphabetic superscripts across columns indicate significant pair-wise differences.
[1]from birth to discharge using Patel geometric method

[2]Ratio= $\frac{\text{Observed serum zinc level within first 48 hours of life}}{\text{Expected serum zinc level for GA using quadratic regression}}$

Expected serum zinc level using quadratic regression = 5.63727 -0.26622 x GA +0.00370 x GA^2

[3] low serum zinc: <0.74 mcg/mL; low vitamin D: <30 ng/mL
[4] NIH consensus
Abbreviations: ROP, retinopathy of prematurity; OR, odds ratio; CI, confidence interval; PMA, postmenstrual age; GA, gestational age
There was no difference in race/ethnicity or sex between the two groups.

Supplementary Table 5.3 Multivariate analysis of ROP in both epochs among infants < 33 weeks excluding comfort care only, n=862

Variable	B coefficient (standard error)	B coefficient Bootstrap CI	aOR (95% CI)	*P* value
Gestational age (by week)	-0.202 (0.073)	-0.345, -0.074	0.817 (0.718, 0.931)	0.002
Birthweight (by 250 grams)	-0.281 (0.105)	-0.500, -0.080	0.755 (0.617, 0.924)	0.006
Stage of bronchopulmonary dysplasia (NIH consensus definition)	0.866 (0.165)	0.581, 1.234	2.377 (1.801, 3.138)	<0.001
Any growth failure pattern	0.728 (0.220)	0.284, 1.166	2.071 (1.377, 3.114)	<0.001
Constant	5.395 (1.795)	2.259, 9.042		<0.001

Stepwise logistic regression analysis with bias-corrected accelerated 95% confidence intervals of the B coefficients was performed using 1000 bootstrap samples with SPSS: n=216 cases with ROP among 862 infants; Nagelkerke R^2=0.472; Hosmer and Lemeshow test <0.001; AUC=0.878 (95% CI 0.854, 0.902); Youden's index 0.693 (predicted probability 0.140, sensitivity 0.907; specificity 0.686)

Not significant: small for gestational age, sex, race/ethnicity, epoch, any zinc level <0.74 mcg/mL, maximum respiratory support, surfactant, severe BPD, RDS, documented sepsis; rate of weight gain was excluded because it was missing in 34 infants.

Abbreviations: aOR, adjusted odds ratio; CI, confidence interval; ROP, retinopathy of prematurity; AUC, area under the curve

Supplementary Table 5.4 Multivariate analysis of ROP in Epoch-2 among infants < 33 weeks excluding comfort care only, n=329

Variable	B coefficient (standard error)	B coefficient Bootstrap CI	aOR (95% CI)	*P* value
Gestational age (by week)	-0.371 (0.136)	-0.649, -0.136	0.690 (0.541, 0.881)	0.002
Birthweight (by 250 grams)	-0.522 (0.221)	-0.960, -0.152	0.593 (0.408, 0.863)	0.008
Stage of bronchopulmonary dysplasia	0.736 (0.330)	0.127, 1.714	2.089 (1.233, 3.538)	0.005
Lowest serum zinc level (mcg/mL)	-3.181 (1.140)	-5.366, -1.124	0.042 (0.006, 0.306)	0.006
Constant	13.970 (3.726)	7.120, 22.624		<0.001

Stepwise logistic regression analysis with bias-corrected accelerated 95% confidence intervals of the B coefficients was performed using 1000 bootstrap samples with SPSS: n=86 cases with ROP among 329 infants; Nagelkerke R^2=0.621; Hosmer and Lemeshow test P=0.334; AUC=0.928 (95% CI 0.899, 0.957); Youden's index 0.716 (predicted probability 0.153, sensitivity 0.942, specificity 0.774).

Not significant: small for gestational age, sex, race/ethnicity, any growth failure pattern, any zinc level <0.74 mcg/mL, low serum zinc or vitamin D level, respiratory distress syndrome, surfactant, maximum respiratory support; rate of weight gain was excluded (missing in some infants).

bronchopulmonary dysplasia, duration of continuous positive airway pressure, duration of ventilation, maximum respiratory support, documented sepsis

Abbreviations: aOR, adjusted odds ratio; CI, confidence interval; ROP, retinopathy of prematurity; AUC, area under the curve

Supplementary Table 5.5. Lowest serum zinc level vs severity of ROP in Epoch-2 among infants <29 weeks gestational age excluding comfort care only

	**No ROP N=49**	**Mild/moderate ROP N=55**	**Severe ROP N=18**	***P* value**
Gestational age (weeks)	26.9 (25.1, 28.0) [a]	26.4 (25.3, 27.9) [a]	24.3 (23.6, 25.09) [b]	0.003
Birthweight (grams)	960 (711, 1128) [a]	830 (707, 1020) [a]	725 (550, 803) [b]	0.012
Lowest serum zinc level (mcg/mL)	0.73 (0.65, 0.93) (n=38)	0.66 (0.60, 0.75) (n=55)	0.64 (0.56, 0.82) (n=18)	0.031
Serum zinc and vitamin D level [1]				<0.001*
Neither measured	11 (22.4%) [a]	0 (0%) [b]	0 (0%) [b]	
Both or either normal	15 (30.6%)	13 (23.6%)	2 (11.1%)	
Low serum zinc level	17 (34.7%)	28 (50.9%)	8 (44.4%)	
Low serum vitamin D level	3 (6.1%)	4 (7.3%)	3 (16.7%)	
Both low	3 (6.1%) [a]	10 (18.2%) [ab]	5 (27.8%) [b]	
Stage of BPD (NIH consensus)				<0.001*
None	40 (82%) [a]	25 (45%) [b]	0 (0%) [c]	
Mild	6 (12%)	9 (16%)	5 (28%)	
Moderate	1 (2%) [a]	10 (18%) [b]	7 (39%) [b]	
Severe	2 (4%) [a]	11 (20%) [b]	6 (33%) [b]	

Values are mean±SD (n) or number (%).

[1] Low serum zinc level: <0.74 mcg/mL; low serum 25 OH vitamin D level: <30 mcg/mL

Kruskal-Wallis test or *Fisher's exact test followed by pair-wise comparisons with Bonferroni correction.

Different alphabetic superscripts across columns indicate significant pair-wise differences.

Abbreviations: ROP, retinopathy of prematurity; BPD, bronchopulmonary dysplasia

Note: all infants with severe ROP had a GA <29 weeks

Supplementary Table 5.6. Internal validation of model of retinopathy of prematurity vs any low serum level of zinc or vitamin D in Epoch-2 among infants < 29 weeks excluding comfort care only, n=122

Variable	Mild/moderate ROP vs no ROP	*P* value	Severe ROP vs no ROP	*P* value
Adjusted odds ratio (95% CI)	4.769 (1.663, 13.675)	0.004	13.584 (1.835, 100.56)	0.011
Beta coefficient (95% CI bootstrapping)	1.562 (0.591, 3.016)	0.002	2.609 (0.259, 21.982)	0.026

Multinomial regression analysis of ROP vs any low serum level of zinc or vitamin D, adjusted for gestational age, birthweight (by 250 g increments) and stage of bronchopulmonary dysplasia was performed using 1000 bootstrapping samples; Nagelkerke pseudo R^2=0.543, goodness of fit deviance *P*=0.999; 66% accuracy
Variables included were those used in Table 5.5, except for the lowest serum Zn level to avoid collinearity.
Abbreviations: ROP, retinopathy of prematurity; CI, confidence interval

Supplementary Table 6. Validation of results extracted from the NICU database among infants born in 2022-2023

**Variable**	**N**	**Intraclass correlation coefficient**	**Cohen kappa**
Gestational age (weeks and days)	384	0.972	
Birth weight (grams)	384	0.972	
Necrotizing enterocolitis	364		1.000
Spontaneous intestinal perforation	374		1.000
Severe intraventricular hemorrhage in extremely low gestational age neonates	201		0.976

Table 7. Morbidities and mortality in Epoch-2 versus zinc level among infants born at < 33 weeks of gestational age,

	**Serum Zinc Levels**			
**Variable**	**At least 1 low N=153**	**Normal (0.74-1.46 mcg/mL) N=143**	**At least 1 high N=29**	***P*-value**
Gestational age, weeks	25 (27, 31)[a]	31 (29, 32)[a]	27 (26, 31)[b]	<0.001
Severe bronchopulmonary dysplasia				0.56
22-28 weeks gestational age	11/60 (18.3)	4/32 (12.5)	3/19 (15.8)	
29-32 weeks gestational age	1/97 (1.0)	0/111 (0)	0/10 (0)	
Severe intraventricular hemorrhage				0.07
22-28 weeks gestational age	7/60 (11.7)	5/32 (15.6)	5/19 (26.3)	
29-32 weeks gestational age	1/97 (1)	3/111 (2.7)	0/10 (0)	
NEC stage II or greater				0.53
22-28 weeks gestational age	6/60 (10)	3/32 (9.4)	0/19 (0)	
29-32 weeks gestational age	2/97 (2.1)	6/111 (5.4)	0/10 (0)	
Retinopathy of prematurity				<0.001
22-28 weeks gestational age	42/60 (70)	16/32 (50)	15/19 (78.9)	0.07
29-32 weeks gestational age	8/97 (8.2)	5/111 (4.5)	0/10 (0)	0.52
Culture negative late-onset sepsis				1.00
22-28 weeks gestational age	8/60 (13.3)	7/32 (21.9)	2/19 (10.5)	
29-32 weeks gestational age	4/97 (4.1)	3/111 (2.7)	0/10 (0)	
Death during first hospitalization				0.02
22-28 weeks gestational age	1/60 (1.7)[a]	5/32 (15.6)[b]	0/19 (0)[ab]	0.02
29-32 weeks gestational age	0/97 (0)	4/111 (3.6)	0/10 (0)	0.28

Values are median (interquartile range) or number (%).
Kruskal-Wallis test or Fisher's exact test followed by pair-wise comparisons (with Bonferroni correction for Fisher's exact test)
Different alphabetic superscripts across columns indicate significant pair-wise differences.

Supplementary Figure 1. Serial serum zinc levels vs postmenstrual age and postnatal age by gestational age group in the growth cohort in Epoch-2

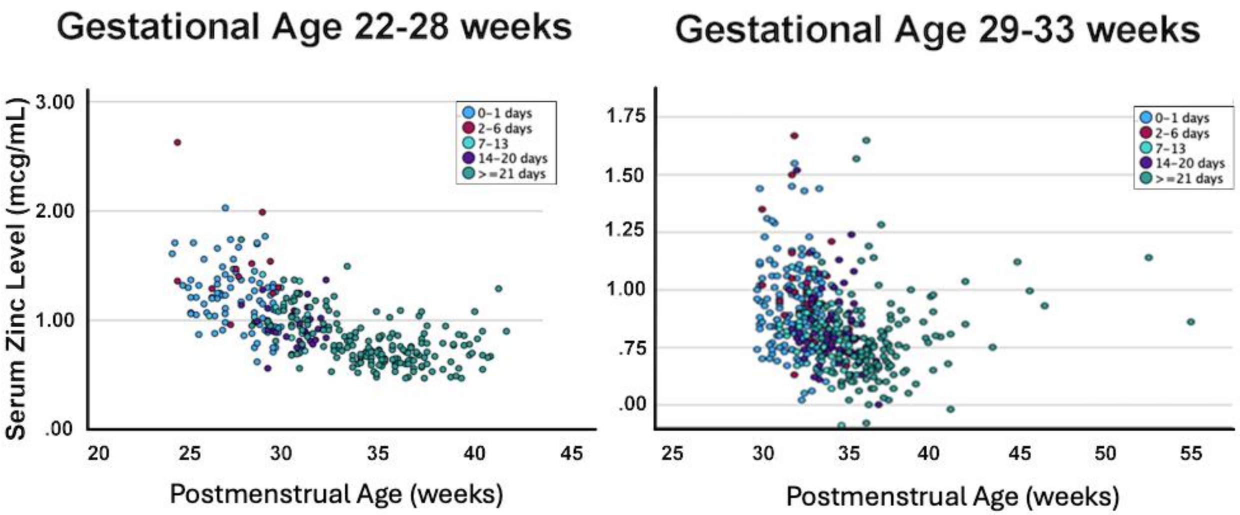

Supplementary Figure 2. Receiver operating curves and calibration curves for the two models predicting retinopathy of prematurity in 329 preterm infants < 33 weeks gestational age in Epoch-2, excluding those on comfort care only

2a. There was no significant difference in area under the curve of the receiver operating curves of the two models (contrast =-0.0128, 95% confidence interval -0.0272, +0.00166, P=0.08).
For model 1 including gestational age, birthweight, stage of bronchopulmonary dysplasia, and any growth failure pattern the area under the curve (AUC) from Receiver Operator Curve (ROC) was 0.9149. The Hosmer-Lemeshow test had a *P*=0.021.
For model 2 including gestational age, birthweight, stage of bronchopulmonary dysplasia and lowest serum zinc level the AUC was 0.9277. The Hosmer-Lemeshow test had a *P*=0.334.

2b. The calibration curve for model 1 was not as linear as 2c, the calibration curve for model 2.

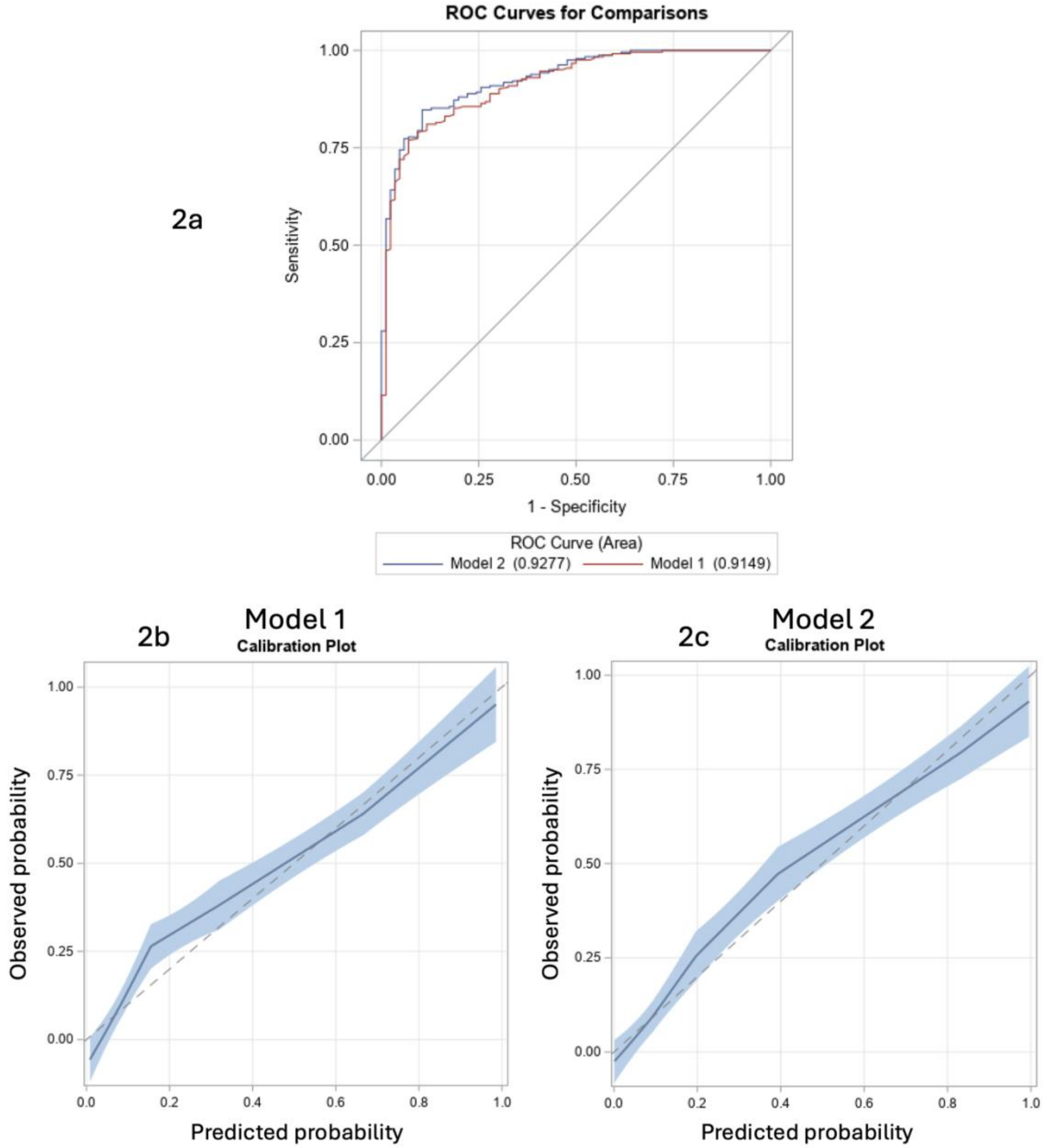

Supplement: Supplementary file 1 — Supplementary Material [file 41390_2025_3935_MOESM1_ESM.pdf]
